# Effect of Stock Plant Growing Medium and Density upon a Cutting Propagation System for Tea Tree, *Melaleuca alternifolia*

**DOI:** 10.3390/plants11182421

**Published:** 2022-09-16

**Authors:** Gail E. Lowe, Mervyn Shepherd, Terry J. Rose, Carolyn Raymond

**Affiliations:** Faculty of Science and Engineering, Southern Cross University, P.O. Box 157, Military Rd, Lismore 2480, Australia

**Keywords:** adventitious rooting, coconut fibre, planting density, stock plant management, vegetative propagation

## Abstract

To offer a viable alternative to seedling deployment of tea tree, clones will require the development of an efficient, robust, and vegetative propagation system for the large number of plants needed for plantations (i.e., typically 33,000 plants/ha). This study investigated the productivity of an intensive management system for tea tree stock plants and rooted cuttings grown in a subtropical environment (Lismore, NSW, Australia). Three stock plant densities (30, 100, and 200 plants/m^2^) were tested in coir and potting mix media (consisting of peat+perlite+vermiculite), with 11 settings of cuttings undertaken between April 2019 and March 2020. All stock plants in each media type survived 11 harvests and remained productive; however after 13 months, many plants in the coir media, appeared chlorotic and showed symptoms of iron deficiency. Rooting and cutting survival rates using the mini cutting technique were high, ranging from a maximum mean monthly setting value of 87.7% ± 4 at 84 days post-setting in potting mix, to a minimum of 80.4% ± 3.7 in coir. The most productive treatment was at high stock plant density in potting mix which had the potential to produce 13,440 plants/year/m^2^. Overall coir appeared less productive, but the pattern of difference among treatments was similar. For the highest system productivity, it is recommended to grow stock plants in potting mix at high densities and modulate temperatures to between 18 °C and 28 °C. Late spring and early summer were the best time for harvesting and setting tea tree mini cuttings in the subtropics.

## 1. Introduction

Tea tree, *Melaleuca alternifolia* (Maiden & Betche) Cheel, is grown in plantations to produce an essential oil sold throughout the world for use in therapeutic and cosmetic products. The tea tree industry in Australia has developed around plantations of genetically improved seedlings produced in seed orchards [1,2].

Although the benefits of using clones to produce highly productive and uniform lines of tea tree were demonstrated in experiments in the 1990s, and there has been some adoption of clones on a small scale for commercial production [1,3], the development of a large-scale commercially viable clonal production system remains elusive [3]. A key challenge for cloning of tea tree is the development of a cost-effective system to produce the large volume of plants required for high planting densities in plantations, typically 33,000 plants per hectare [4]. Due to the large labour component of vegetative propagation, cost per clonal propagule is typically 5 to 10 times that of a seedling, which presents a significant impediment to the use of clones compared to seedlings at the time of plantation establishment [3]. Nursery production systems would also need to generate an estimated 3 million clonal propagules a year to cater for anticipated demands for industry expansion and refurbishing existing plantations with new lines.

Other constraints for a commercially viable clonal propagation system for tea tree are the management of maturation effects associated with vegetative propagation of woody plants [5,6] and production of a propagule amenable to machine planting. Machine planters require an upright, single stem plant with a small crown. Recent research has shown that these challenges are surmountable when appropriate cutting techniques and appropriate maturation and propagation management are used [5,7]. However, the simplicity, reliability, and low cost of seedling propagation systems for tea tree presents a challenging benchmark for the more labour intensive and technically demanding clonal systems to match.

One major factor influencing the success of propagation by rooted cuttings is the nutrition and health of the stock plants. Optimising stock plant nutrition not only ensures maximum growth and productivity, but scion must also be primed with sufficient nutrition prior to harvest of cuttings, because there is little nutrient uptake through the cut stem before roots have formed [8,9]. Additionally, of consideration is the growing media stock plants are grown in [10]. The most productive substrates usually consist of a mixture of components with high organic content that favour root development, ensure nutritional balance, and have a high capacity for water retention [11].

Competition for resources can also impact on stock plant development and growth [12]. Consequently, stock plant density is a key factor in the productivity of the clonal system [8]. How this will play out under different stocking scenarios is not necessarily foreseeable. Where nutrient supply is not limited, plant growth and therefore cutting production per plant may be maximised [13]. However, while the number of suitable scions for harvest per plant is likely to be maximised under lower plant densities, productivity at a system level may be higher per square metre with higher plant densities even though fewer cuttings may be harvested per plant [14]. A further consideration is the quality of the scion and the subsequent rooting rates of cuttings. Stock plants grown at higher densities may also be subject to nutrient stress due to competition, which may accelerate maturation, which in turn may impact the woodiness and quality of the cuttings [12,15]. Because light is supplied from above, plant density can also impact on plant development. Taller plants will benefit directly from increased photosynthetic rates and indirectly by reducing the growth of neighbours via shade, causing variability among stock plants which may reduce the number of mini cuttings available for harvest [12,16,17].

Advances in propagation methods of *Eucalyptus* species have culminated in the development of a super-intensive system for the management of eucalypt stock plants [8,14]. These mini cuttings systems can produce up to 25,000 high quality propagules per square metre of stock plants per year [8,14]. The aim of the present study was to explore the potential of adopting similar large scale, intensive clonal production systems for tea tree.

This study investigated three factors thought to be key to the efficacy of the production systems: stock plant density, growth media, and clonal line. This study tested whether the type of growing media and tea tree stock plant density influence stock plant maturation and productivity, and thus the number and quality of rooted mini cuttings. The study was conducted over a 12-month period, in order to examine fluctuations in the system productivity due to season and long-term health of the stock plant, as well as to allow estimation of annual productivity rates.

## 2. Results

### 2.1. All Stock Plants Survived and Remained Productive but a Fe Deficiency Likely Reduced Productivity of Coir

**Figure 1:**
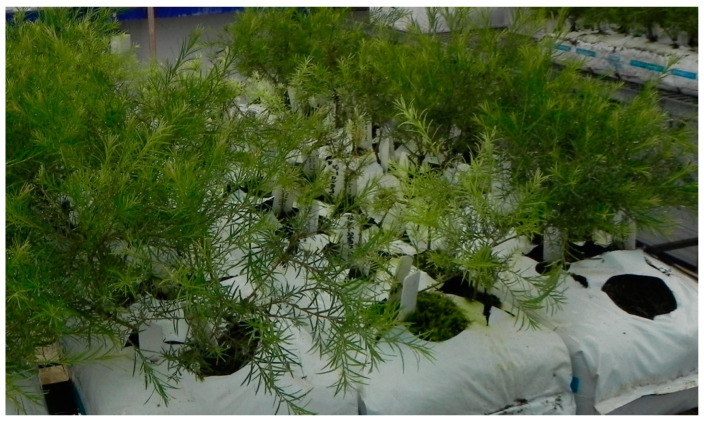
Plants of tea tree (*M. alternifolia*) showing signs of leaf chlorosis in the middle bag after 12 months of cultivation (medium density coir).

All results are reported based on data pooled over the two clones of tea tree as there was no significant difference between clones for stock plant or cutting attributes (data not shown). All stock plants survived over the 12-month period of the experiment, and 11 harvests, but stock plants grown in coir developed a chlorotic appearance, despite regular fertilisation (Figure 1). This first appeared after 6 weeks and deteriorated over the course of the experiment. Shoot tip tissue analysis in these plants indicated significantly (*p* < 0.001) lower iron (Fe) concentrations relative to plants growing in potting mix (Table 1). Shoot tip nitrogen (N) levels observed in this study were within the range previously shown to be sufficient to achieve high rooting on mini cuttings of tea tree [7,8]. The number of stock plants with chlorotic appearance, was significantly higher in those growing at low density, than those growing at medium or high density in coir (*p* < 0.001). After 360 days, 80% of the plants in coir at low density were affected (Table 1). As a consequence of this probable nutrient deficiency in one media, it was decided not to statistically compare media differences and they were analysed separately.

### 2.2. The Effect of Potting Media

#### 2.2.1. Stock Plant Productivity

In potting mix, seasonal effects (assessed at each setting date) significantly (*p* < 0.05) affected all four dependent variables (Table 2). Stock plant density affected stock plant height and the number of cuttings produced but there were no significant interactions between density and setting date (Table 2).

Overall stock plants grew most vigorously at low density in potting mix, with significantly less growth occurring in plants at high density (mean height ± SE for low, medium, and high densities; 21.1 ± 0.5 cm, 19.3 ± 0.5 cm, and 18.7 ± 0.6 cm, respectively (Table 3). Mean cuttings per plant (averaged across harvest periods) was highest in plants grown at low density (means ± SE low, medium, and high values, 8.9 ± 1.0, 7.1 ± 0.6, and 5.6 ± 0.3 cuttings/plant/month) (Table 3). However, on a per square metre basis, productivity was highest at the highest stocking rates at 13,440 cuttings/m^2^/year (Table 4).

#### 2.2.2. Cuttings Rooting and Survival

**Figure 2:**
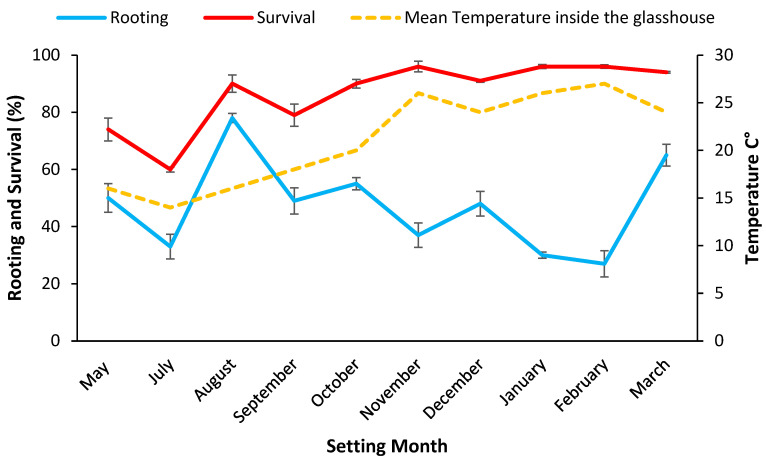
Setting date means (i.e., data pooled over densities) for rooting rate % 28 days post setting and survival rate % 84 days post setting for mini cuttings from stock plants grown in potting mix plotted with the mean temperature inside the glasshouse during the setting months. Bars represent SE.

Survival of mini cuttings after 28 days derived from stock plants grown in potting mix was high across all setting dates and ranged from 93.3% to 100%. There was no difference in mean rooting at this time (Table 3). At 84 days post setting, mean survival for a setting date ranged from 60% to 97% (Figure 2) with the highest survival recorded at high density for the 5th setting during August (99.5% ± 0.4). The time of year cuttings were set had a significant (*p* < 0.001) influence on the rooting rate (28 days post setting) and survival (84 days post setting) of the mini cuttings, but the effect of density was not significant (Table 3).

### 2.3. Coir

#### 2.3.1. Stock Plant Productivity

In general, the patterns evident in potting mix, were also seen in coir. All stock plants survived, stock plant density affected the number of cuttings, but not stock plant height (Table 2). The high-density coir produced fewer cutting per month, than the medium and low treatments, which were not different to each other (means SE low, medium, and high values; 6 ± 0.5, 5.6 ± 0.4, and 4.7 ± 0.3 cuttings per month, respectively) (Table 3). Like potting mix, the highest productivity rate for cutting production on a per plant basis in coir was in the low plant density (Table 3). Additionally, like potting mix, the highest system productivity was found using the highest stocking rate, 11,280 cuttings/m^2^/year, compared to 6720 and 2160 cuttings/m^2^/year, for the medium and low-density treatments, respectively (Table 4).

Setting date also significantly (*p* < 0.01) influenced all four dependent variables, but stock plant density significantly influenced the number of cuttings (*p* < 0.05) and survival (*p* < 0.01) in this case (Table 2 and see below in Section 2.4). There was one significant (*p* < 0.001) density and setting date interaction effect for cutting survival at 84 days, when stock plants were grown in coir (Table 2).

#### 2.3.2. Cutting Rooting and Survival

As with potting mix, all cuttings appeared alive when removed from the propagation enclosure after 28 days. Rooting rate of cuttings was not significantly different for coir therefore only results for cutting survival are discussed (Table 3). Survival in the high and medium density treatments were not different but were both significantly (*p* < 0.01) higher than the low treatment (Table 3).

### 2.4. Seasonal and Interaction Effects (on Stock Plant Productivity, and Cutting Rooting and Survival)

#### 2.4.1. Stock Plants Grown in Potting Mix

Maximum stock plant growth (23.5 ± 1.0 cm) occurred during the spring months of September and November 2019, and growth rates were generally lower in mid-winter and over the hot summer months (Table A2). When rooting was assessed at 28 days (data pooled across density for all settings), significantly (*p* < 0.001) more cuttings set during August 2019 had formed roots (68.9% ± 1.6) compared to the hotter months (<46%), suggesting the milder weather favoured early rooting (Figure 2). Survival was significantly (*p* < 0.001) higher for mini cuttings set during November 2019, January, and February 2020 (96%) than at other setting dates (Figure 2).

#### 2.4.2. Stock Plants Grown in Coir

The highest stock plant growth in the coir was also measured during spring 2019 in the months of September (21.3 ± 0.4 cm) and November (20.7 ± 0.3 cm) (Table A3). When growth during the first month after planting was disregarded (April 2019), significantly (*p* < 0.001) less growth occurred during winter 2019 in July (15.9 ± 0.2 cm) and summer 2019/20 (16.3 ± 0.6 to 17 ± 0.2 cm) than other setting dates (Table A3).

**Figure 3:**
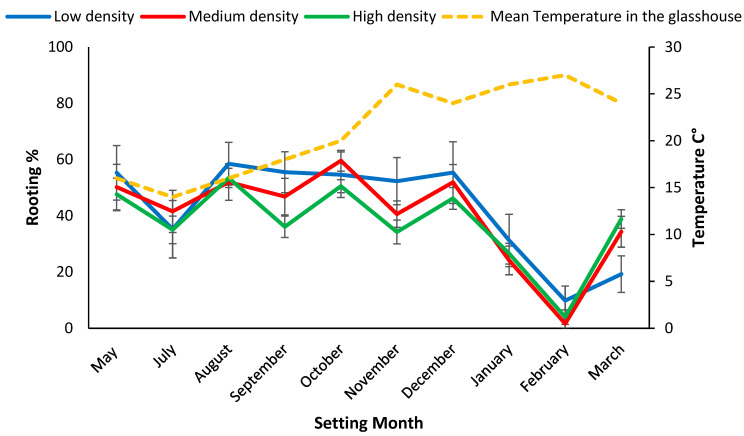
Setting date means (i.e., data pooled densities) for rooting rate% 28 days post setting for mini cutting from coir and mean temperature inside the glasshouse during setting months. Bars represent SE.

**Figure 4:**
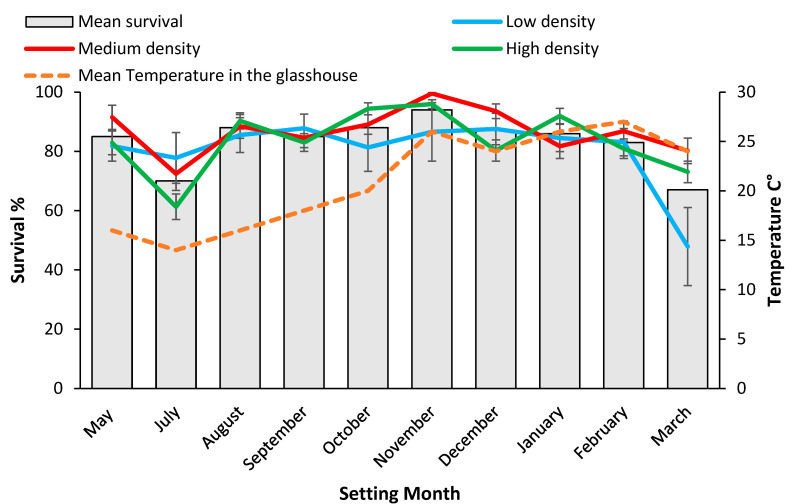
Setting date means for survival rate% 84 days post setting for mini cutting from coir and mean temperature inside the glasshouse during setting months. Values for the mean (pooled over densities) as well as each density level are shown. Bars represent SE.

When rooting was assessed at 28 days, significantly (*p* < 0.001) more cuttings set during August and October 2019 had formed roots (55%) compared to the hotter months (<46%) (Figure 3). Survival after 84 days was significantly (*p* < 0.001) higher for mini cuttings set during November 2019, 94% than other setting dates (Figure 4). Mini cuttings set in March 2020, (67% ± 9.8) and mid-winter, (70.5% ± 4.9) (July 2019) had the lowest average survival rate (Figure 4). Survival from the low-density treatment of coir set in March 2020 of 47.9% ± 13.2 was an outlier, and significantly less than for either the high (73.1% ± 3.6) or the medium density treatments (80.2 ± 4.3) (Figure 4). This outlier drove the significant density *x* setting date interaction effect for survival in coir and was probably related to the high proportion (80%) of chlorosis in these plants (Table 1 and Figure 4).

## 3. Materials and Methods

### 3.1. Propagation Environment

The stock plants were grown in a rooftop glasshouse at Southern Cross University, Lismore Campus, NSW, Australia (28.82° S 153.29° E), from February 2019 to March 2020. The climate of the region is subtropical with average daytime and night-time temperatures inside the glasshouse during the study period varying between 25 °C and 13 °C with a minimum of 2.2 °C (during July, winter) and a maximum of 43.4 °C (during January, summer). Rooting of cuttings was carried out (over 28 days) in a polythene enclosure inside a separate glasshouse where the mean monthly temperatures inside the glasshouse housing the chamber ranged between 8.5 °C (night-time) and 38.5 °C (daytime) (Table 5) (see below for further description of methods).

### 3.2. Stock Plant Origins

Stock plants were produced by serial propagation (i.e., repeated propagation over several cycles or from shoots from other recently rooted cuttings [15]) between October and December 2018 using the mini cutting technique [5]. The cuttings came from stock plants (approximately 2 years old) propagated through several rounds of serial propagation by staff at Queensland Department of Agriculture and Fisheries at a facility in Gympie QLD, Australia, using the nodal stem cutting technique. Two lines differing in propagation rates using the nodal stem cutting technique (i.e., cutting made from semi lignified stem section), a high rooting line (93%) and a low rooting line (53%), were chosen for investigation [2]. After rooting, the mini cuttings were kept in a glasshouse with micro sprinkler irrigation and fertilised once a week with 5 L/m^2^ of Canna Classic Vega (A and B) (Canna Australasia, Subiaco, WA, Australia) at the manufacturers recommended concentration.

### 3.3. Experimental Design and Management of Stock Plants

The trial was set up with a factorial design to test for the effect of stock plant density, growing medium, or clone, on stock plant productivity (number of cuttings/plant) and rate of cutting rooting and survival. Plants were grown in “grow bags” 100 cm × 20 cm × 10 cm (Dutch Plantin, Boekel, NL) that came pre-packed with coir. For this experiment half of the grow bags were repacked with a potting mix consisting of (2:1:1) sphagnum peat moss (Theriault & Hachey Peat Moss Ltd., Baie-Sainte-Anne, NB, CA) perlite and vermiculite (Australian Vermiculite Co Pty Ltd., Carole Park, QLD, Australia), treated with 1 g dolomite (Mudgee Dolomite & Lime Ltd., Mt. Knowles, NSW, Australia) per L media to neutralise the pH (no added nutrients) to allow testing of a second medium. Both media had been chosen because their physical characteristics had been shown to encourage root development and superior growth of tea tree cuttings [11,18]. The organic content and pH of the media used in this study are presented in Table A1.

**Figure 5:**
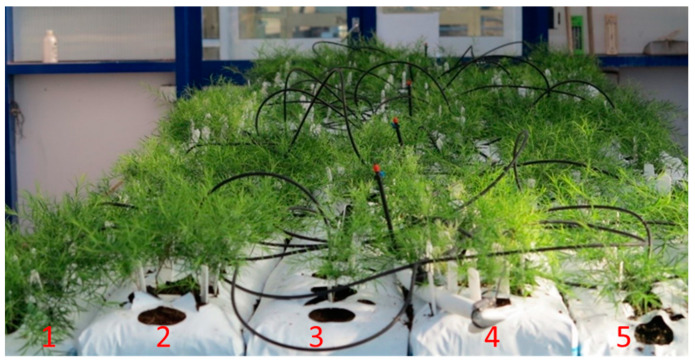
Stock plant growing conditions. Bag 1—(far left) pot mix high density (40 plants per bag; 200 plants/m^2^), bag 2—coir medium density (20 plants per bag; 100 plants/m^2^), bag 3—coir low density (6 plants per bag; 30 plants/m^2^), bag 4—coir high density (40 plants per bag; 200 plants/m^2^), and bag 5 —potting mix low density (6 plants per bag; 30 plants/m^2^) pot mix.

The experiment was arranged as a randomised complete block design with two growing media (coconut fibre (coir) or potting mix) × 3 densities; (1) 30 plants/m^2^ (low; one row 15 cm apart), (2) 100 plants/m^2^ (medium; two rows 10 cm apart), or (3) 200 plants/m^2^ (high; four rows 5 cm apart), with 5 replicates per treatment. The two clones were randomly planted within each bag. In total the experiment consisted of 660 plants (Figure 5).

The stock plants were transferred to the glasshouse in early February 2019 to acclimatise for 21 days before being planted into the experimental layout in grow bags. After planting, stock plants were trimmed to a height of 12 cm to have a common baseline for measuring growth and to encourage flush regrowth. The stock plants received drip irrigation twice per day at an average flow rate of 6 L/m^2^/day and were hand fertilised once a week with 1 L of Canna Classic Vega (A and B) (Canna Australasia, Subiaco, WA, Australia) per bag. No treatments were undertaken for insect or fungal pests at planting.

### 3.4. Scion Collection and the Effects of Setting Date

Beginning in April 2019, cuttings were harvested every 30 days (referred to as “setting date”) over a 12-month period to evaluate the effect of season on potential production of mini cuttings and the effect on rooting ability over a whole year. In total, 11 setting dates were evaluated: 30, 60, 120, 150, 180, 210, 240, 270, 300, 330, and 360 days after initial shoot pruning. No cuttings were collected for setting at the third cycle (90 days; June 2019) due to a mite infestation. Stock plants were treated with Summer Oil^®^ (Sacoa Pty. Ltd., Claremont, WA, Australia) at this time. Within each treatment, a set of plants were randomly selected for sampling throughout the course of the experiment. Four, two, and one plant per clone, per replicate, were selected from the high-, medium-, and low-density treatments, respectively. All shoots >5 cm in length were collected from each as prospective scion. Shoots less than 5 cm in length were left on the stock plants for subsequent collections. After scion collection, all stock plants, including those not designated for scion collection, were trimmed to a height of 15 cm at every setting date so that they were treated equally.

At each setting date, the survival and health of the stock plants, height (as a surrogate for growth), and the number of mini cuttings collected per stock plant sampled were recorded. Tip cuttings were also collected from the stock plants after 12 months to determine the nutrient content of the stock plants and the potential effect of repeated harvesting. Tip cuttings for tissue nutrient analysis were dried at 40 °C for 2 weeks in a drying room at Southern Cross University to initially stabilise the tissue for storage. Tissue nutrient analysis was performed by Southern Cross University Environmental Analysis Laboratory. Prior to analysis, tissue was further dried to 70 °C for 24 h to allow values to be expressed on a dry weight basis. The concentration of N was measured using a LECO TruMAC CNS analyser. Iron concentration was measured using an APHA 3125 ICP-MS after a 0.2 g subsample of plant tissue was digested in aqua regia in a MARS Xpress microwave oven (CEM Corporation, Matthews, NC, USA).

### 3.5. Propagation of Mini Cuttings

Cuttings were collected from the stock plants in the morning and set on the same day as collection. The cuttings were prepared and set following the technique described in Lowe et al. (2019). Briefly, scion was trimmed to 3 cm, and lower leaves removed. The terminal leaves were trimmed above the apical bud. The stem of each cutting was dipped into a commercial rooting auxin powder (Yates Plant Cutting Powder, Yates, Padstow, NSW, Australia), then set into a 9 × 22 cell (198 cells) seedling tray (total volume of 19 cm^2^) (Plantway Ltd., Modesto, CA, USA).

Rooting medium consisted of 60% sphagnum peat moss (Theriault & Hachey Peat Moss Ltd., Baie-Sainte-Anne, NB, CA) and 40% perlite (Australian Vermiculite Co Pty Ltd., Carole Park, QLD, Australia), treated with 1 g dolomite (Mudgee Dolomite & Lime Ltd., Mt. Knowles, NSW, Australia) per L media to neutralise the pH (no added nutrients).

The set cuttings were grown in a polythene enclosure (0.8 × 1.8 m) (Sage Horticulture Pty, Hallam, VIC, Australia) with a balance arm mister to maintain humidity (>80%) and underbed heating set at 22 °C. A USB data logger (Lascar Electronics, Adel., SA, Australia) monitored air humidity and temperate during propagation. The cuttings remained in the enclosure for 28 days, before being removed and placed on a bench inside the glasshouse. The cuttings stayed on the bench for a further 56 days, watered by mist irrigation for 1 min every 6 h.

### 3.6. Propagule and Root Assessment

The rooting percentage of the cuttings for each treatment was determined at 28-, 56-, and 84-days post-setting. Rooting was assessed by carefully removing cuttings from the rooting media and recording whether roots of any length were present or absent.

### 3.7. Statistical Analysis

Initially, a 4-way Analysis of Variance was used to test for the effect of planting density, media type, clone, and setting date on each of the five dependent variables: stock plant growth (height), survival, and cutting productivity, rooting rate, and survival of the cuttings. The effect due to clone line was not significant for any main or interaction factor, therefore data were pooled over the two clonal lines for further analysis (analysis not shown). Three-way ANOVA on pooled data was then used to test for the main effects of density, media, and setting date, and their interactions on each of the five dependent variables.

Where the F test for a main effect was significant (*p* ≤ 0.05), post hoc testing among treatments was carried out using Duncan’s multiple range test. All statistical analysis was performed using Genstat, Release 19.1 [19]. Percentages were transformed with the ‘arcsin’ square root function before ANOVA but have been back-transformed to percentages for reporting here. For the purpose of transformation and data analysis, 1 month was considered equivalent to a 30-day period. System productivity rates were calculated using the mean number of cuttings per plant per month over 11 settings for each planting density in each type of growing medium, then multiplying by 12 for an annual rate.

## 4. Discussion

This study showed the potential for cloning tea tree using elements adopted from intensive production systems described for eucalypts [8]. High propagule productivity and high survival of tea tree stock plants was achieved after monthly scion harvests over 12 months inside a glasshouse.

### 4.1. Tea Tree Stock Plants Are Amenable to Intensive Management

Tea tree stock plants survive and grow well under intensive management, so long as adequate nutrition is provided. High densities impact growth as expected but nonetheless, provide the highest productivity rates on a square metre basis. There does not seem to be a penalty in terms of rooting of mini cuttings.

Healthy and vigorous stock plants will improve the success of cutting production by the mini cutting technique [8]. The high survival rate of the stock plants (100%) and sustainment of productivity over 12 months found in this study in both media and at the three densities tested suggests tea tree will be amenable to intensive stock plant management. Stuepp et al. [11], also recorded high survival rates (>94%) for tea tree stock plants growing as a mini clonal garden in a coir and peat medium. High stock plant survival (>90%) has also been reported for *Eucalyptus* [20,21]. Loss of stock plants was observed in the winter months during these studies, which may have been also subject to more intensive cropping.

Tea tree stock plants growing in the potting mix grew vigorously throughout the four seasons of the year, suggesting the composition and nutritional conditions provided were adequate. Tea tree stock plants growing in the coir, however, showed a reduction in vigour over time, but no plant death. The gradual decline in stock plant health in the coir was unexpected as grow bags are a commercial substrate consisting of 100% coconut fibre and are used regularly to grow numerous crops [22,23]. It has been suggested that coir fibre substrates can be low in Fe [22,24] or can become depleted in Fe over time [24] and this appeared to be a factor in our experiment. Coconut-derived substrates can have high pH [25] which also reduces Fe bioavailability [26]. Values for pH were low for both media in this study, ranging from 4.7 in the potting to 6.9 in the coir, which was within the recommended range for tea tree [27]. The shoot tip Fe concentrations were consistent with low bioavailable Fe in the coir and may be overcome in subsequent studies through supplemental Fe additions.

The frequency of scion harvesting is also an important aspect of stock plant management, not only to meet productivity targets, but to avoid significant decline in stock plant health between collections [28]. Our data suggest harvesting every 30 days is not detrimental to stock plant health in the long term.

### 4.2. System Productivity was Influenced by Planting Density

Planting density significantly influenced the number of cuttings available for harvesting per month for both media, with more cuttings harvested per plant from the low density (15 cm spacing) stock plants (Table 3), consistent with competition for nutrition reducing per plant productivity. However, total output per square metre was higher at high density, around 1100 cuttings/m^2^/month. Therefore, despite the impact of reduced per plant productivity at higher densities, higher stocking rates would be more productive on a per square metre basis, and it may be possible to boost these further with additional nutrition to further ameliorate competition effects. It was possible to have higher rates of productivity in eucalypt systems. Up to 8000 cuttings per month have been achieved in *Eucalyptus benthamii x E. dunnii* stock plants at 10 × 15 cm/m^2^ spacing subjected to 41 consecutive mini cutting collections in one year [29], suggesting further increases in productivity may also be possible in tea tree systems with even higher stock plant density, or cutting frequency.

The highest mean production of mini cuttings was observed during spring, November 2019, for both media. This is not surprising as environmental variations and the characteristics of each season of the year are factors that can influence shoot production in stock plants. Plants are in full vegetative growth during the warmer months, with new bud outgrowth and young juvenile leaves [30]. The seasonal effect was also evident towards the end of summer (February 2020), with the higher temperatures influencing stock plant growth resulting in a decline in available cutting material (Table 3). However, average mini cutting productivity was significantly higher the following month, indicating a resurgence in stock plant growth as the temperature fell and conditions became more favourable at the beginning of March 2020 (autumn). The recovery in growth also suggests the possibility that tea tree is able to maintain juvenility over successive scion collections. Maintaining the juvenile vigour of the stock plant is essential for successful rooting of mini cuttings [8].

### 4.3. Rooting Rates Varied by Setting Season but Not by Genotype

It is generally observed that the highest mean percentage of rooting occurs in the summer months [11]. In our study the highest rooting occurred in mini cuttings set during mid to late summer (96.6%) for potting mix and late spring (94%) for coir. The 28-day maintenance period in the propagation enclosure for rooting on tea tree mini cuttings was adequate, considering the good rooting observed when the mini cuttings were removed. This time period is less than that of 35 and 42 days, recommended for *Eucalyptus* mini cuttings [21]. At the 28-day assessment post setting, the milder temperature favoured rooting, with higher rooting found in mini cuttings set during August 2019 for both potting mix (68.9%) and coir (54.6%) (Figure 2 and Figure 3).

Rooting rates in this study were not affected by genotype. This was unexpected based on the a priori propagation rates as nodal stem cuttings. Since that time, however, evidence from other studies has shown, that a very broad range of genotypes of tea tree will root at very high levels provided appropriate technique (i.e., tip cuttings are preferable to nodal stem cuttings) is used, even when starting with aged ortets [2].

## 5. Conclusions

The high survival and productivity of the tea tree stock plants in the glasshouse demonstrated the feasibility of intensively managing stock plants at high densities to produce high quality propagules. A spacing of 5 cm, 10 cm, or 15 cm between stock plants did not affect the rooting capacity of the mini cuttings and there were no stock plant losses at any planting density.

The potting mix of peat, vermiculite, and perlite proved to be the superior medium for tea tree stock plants; however, with monitoring and nutrient adjustment, coir fibre is also considered to be an appropriate medium for successfully growing tea tree stock plants. Late spring and early summer were the best time for setting tea tree mini cuttings in the subtropics, or climate control should be introduced to modulate the environment.

## Figures and Tables

**Table 1:** Percentage of tea tree (*M. alternifolia*) stock plants showing signs of chlorosis after 360 days with leaf tissue iron (Fe) and nitrogen (N) concentrations. Means followed by the same letter in each column were not significantly different at the 95% confidence level.

Media	Density	Stock Plants Affected	Nutrient Concentration	
		(%)	Fe (mg/kg)	N (%)
*Potting Mix*	Low	0 ^c^	62 ^b^	2.65 ^a^
	Medium	0 ^c^	71 ^b^	2.16 ^a^
	High	0 ^c^	64 ^b^	2.05 ^a^
*Coir*	Low	80 ^a^	37 ^a^	2.82 ^a^
	Medium	22 ^b^	40 ^a^	2.58 ^a^
	High	20 ^b^	39 ^a^	2.80 ^a^

**Table 2:** Significance of main and interaction effects in a 2-way ANOVA for media type.

Parameters				
	Stock Plant Height	No. of Cuttings	Rooting % 28 Days	Survival % 84 Days
*Potting Mix*				
Density	*	**	ns	ns
Setting Date	***	*	***	***
Density x Setting Date	ns	ns	ns	ns
*Coir*				
Density	ns	*	ns	**
Setting Date	***	**	***	**
Density x Setting Date	ns	ns	ns	***

ns = not significant, *** *p* < 0.001, ** *p* < 0.01 and * *p* < 0.05.

**Table 3:** Response of stock plant and cutting parameters to media type and planting densities. Means pooled over setting time are shown with SE. Means followed by the same letter are not significantly different at the 95% confidence level.

Stock Plant Density	Stock Plant Survival (%)	Mean Stock Plant Height (cm)	Mean No. Cuttings/Month	Mean Rooting 28 Days (%)	Mean Survival 84 Days (%)
*Potting mix*					
Low	100	21.1 ± 0.5 ^a^	8.9 ± 1.0 ^a^	43.5 ± 4.1 ^a^	83.3 ± 4.0 ^a^
Medium	100	19.3 ± 0.5 ^b^	7.1 ± 0.6 ^b^	44.3 ± 3.8 ^a^	86.6 ± 4.0 ^a^
High	100	18.7 ± 0.6 ^c^	5.6 ± 0.3 ^c^	46.3 ± 3.8 ^a^	87.7 ± 4.0 ^a^
*Coir*					
Low	100	18.0 ± 0.5 ^a^	6.0 ± 0.5 ^b^	42.7 ± 5.7 ^a^	80.4 ± 3.7 ^a^
Medium	100	17.8 ± 0.6 ^a^	5.6 ± 0.4 ^b^	41.4 ± 5.0 ^a^	86.8 ± 2.4 ^b^
High	100	17.8 ± 0.6 ^a^	4.7 ± 0.3 ^a^	36.9 ± 4.6 ^a^	83.4 ± 3.0 ^ab^

**Table 4:** Productivity of tea tree (*M. alternifolia*) stock plants. Means followed by the same letter are not significantly different at the 95% confidence level.

Stock Plant Density	Plants/m^2^	Mean Cuttings/Month	Average Productivity Cuttings/m^2^	Annual Productivity Cuttings/m^2^/Year
*Potting mix*				
Low	30	8.9 ± 1.0 ^a^	267	3204
Medium	100	7.1 ± 0.6 ^b^	710	8520
High	200	5.6 ± 0.3 ^c^	1120	13,440
*Coir*				
Low	30	6 ± 0.5 ^b^	180	2160
Medium	100	5.6 ± 0.4 ^b^	560	6720
High	200	4.7 ± 0.3 ^a^	940	11,280

**Table 5:** Temperatures inside the glasshouse housing the rooting chamber during the study period.

Year	Season	Minimum Mean Night-Time Temperature (°C)	Maximum Mean Daytime Temperature (°C)
2019	Autumn (March–May)	9.5	28.0
2019	Winter (June–August)	8.5	20.6
2019	Spring (September–November)	21.0	34.5
2019/2020	Summer (December–February)	25.0	38.5
2020	Autumn (March)	11.0	29.0

## Data Availability

Research data are not shared.

## Appendix A

**Table A1:** Organic matter and pH for coir and potting mix components.

	*Coir*	*Potting Mix*		
		Sphagnum Peat Moss	Perlite	Vermiculite
Organic matter	99%	98–99%	-	-
pH	5.8–6.5	3.5–3.8	7.2	8–10

Data sourced from product manufactures.

**Table A2:** Setting date means (i.e., data pooled across densities) for stock plant height before the collection of mini cuttings and stock plant productivity (mean number of mini cuttings per stock plant) for potting mix. Means followed by the same letter show no significance at the 95% confidence level.

Setting Date	Mean Stock Plant Height (cm)	Mean No. Cuttings/Stock Plant
1. April	16.0 ± 0.3 ^a^	4.1 ± 0.2 ^a^
2. May	18.7 ± 0.2 ^bc^	5.7 ± 0.5 ^bcde^
4. July	17.5 ± 0.8 ^b^	4.8 ± 0.2 ^abc^
5. August	19.2 ± 0.5 ^c^	5.8 ± 0.5 ^cde^
6. September	23.5 ± 0.6 ^e^	8.7 ± 1.3 ^f^
7. October	21.1 ± 1.2 ^d^	7.9 ± 0.9 ^f^
8. November	23.5 ± 1.0 ^e^	10.5 ± 2.5 ^g^
9. December	17.7 ± 0.9 ^b^	8.9 ± 1.2 ^f^
10. January	18.4 ± 0.5 ^bc^	7.9 ± 1.4 ^f^
11. February	19.5 ± 1.7 ^c^	6.9 ± 1.6 ^e^
12. March	21.2 ± 0.9 ^d^	8.5 ± 1.4 ^f^

**Table A3:** Setting date means (i.e., data pooled across densities) for stock plant height before the collection of mini cuttings and stock plant productivity (mean number of mini cuttings per stock plant) for coir. Means followed by the same letter show no significance at the 95% confidence level.

Setting Date	Mean Stock Plant Height (cm)	Mean No. Cuttings/Stock Plant
1. April	15.8 ± 0.1 ^a^	3.9 ± 0.4 ^a^
2. May	17.3 ± 0.3 ^cd^	5.1 ± 0.6 ^abc^
4. July	15.9 ± 0.2 ^a^	4.1 ± 0.1 ^a^
5. August	17.9 ± 0.4 ^d^	4.7 ± 0.4 ^ab^
6. September	21.3 ± 0.4 ^g^	6.6 ± 0.7 ^e^
7. October	18.9 ± 0.1 ^e^	6.2 ± 0.5 ^de^
8. November	20.7 ± 0.3 ^f^	7.6 ± 0.8 ^f^
9. December	16.6 ± 0.2 ^bc^	5.8 ± 0.4 ^de^
10. January	17.0 ± 0.2 ^cd^	5.7 ± 0.4 ^cde^
11. February	16.3 ± 0.6 ^ab^	3.9 ± 0.3 ^a^
12. March	18.8 ± 0.1 ^e^	5.9 ± 1 ^bcd^
